# Tilt-structure and high-performance of hierarchical Bi_1.5_Sb_0.5_Te_3_ nanopillar arrays

**DOI:** 10.1038/s41598-018-24872-4

**Published:** 2018-04-23

**Authors:** Ming Tan, Yanming Hao, Yuan Deng, Dali Yan, Zehua Wu

**Affiliations:** 1 Department of Physics, College of Sciences, Tianjin University of Science & Technology, Tianjin, 300457 China; 2 Beijing Key Laboratory of Special Functional Materials and Films, School of Materials Science and Engineering, Beihang University, Beijing, 100191 China; 3 College of Physics and Materials Science, Tianjin Normal University, Tianjin, 300387 China

## Abstract

The uniquely tilted nanopillar array favorably influence carrier and phonon transport properties. We present an innovative interfacial design concept and a novel tilt-structure of hierarchical Bi_1.5_Sb_0.5_Te_3_ nanopillar array comprising unique interfaces from nano-scaled open gaps to coherent grain boundaries, and tilted nanopillars assembled by high-quality nanowires with well oriented growth, utilizing a simple vacuum thermal evaporation technique. The unusual structure Bi_1.5_Sb_0.5_Te_3_ nanopillar array with a tilt angle of 45° exhibits a high thermoelectric performance *ZT* = 1.61 at room temperature. The relatively high *ZT* value in contrast to that of previously reported Bi_1.5_Sb_0.5_Te_3_ materials and the Bi_1.5_Sb_0.5_Te_3_ nanopillar array with a tilt angle of 60° or 90° evidently reveals the crucial role of the unique interface and tilt-structure in favorably influencing carrier and phonon transport properties, resulting in a significantly improved *ZT* value. This method opens a new approach to optimize nano-structure film materials.

## Introduction

Thermoelectric (TE) materials can directly interconvert thermal energy and electrical energy by on the basis of Seebeck effect and Peltier effect. TE energy conversion efficiency is determined by *ZT* = (*S*^2^*σ/κ*)*T*, where *σ*, *S*, *κ*, and *T* are electrical conductivity, Seebeck coefficient, thermal conductivity, and absolute temperature, respectively^1–3^. There exists a strong coupling of TE parameters *κ*, *σ* and *S*. Many research efforts to overcome the conventional *κ*-*σ* and *σ*-*S* trade-off have been made in attempts to obtain a high *ZT* value during recent years^4–7^. Theoretical and experimental analyses have shown that the low-dimensional structure can significantly optimize the transport properties of electrons and phonons, which are to break through the limitation of the electron-phonon coupling and provide an effective pathway past a low-dimensional structure material, such as, a *ZT* value of 3 was reported for the PbSeTe/PbTe quantum dot superlattice, and the record high *ZT* value of 2.4 was achieved for the Bi_2_Te_3_/Sb_2_Te_3_ superlattice. However, One-dimensional nanowires are predicted to exhibit a better TE property than superlattices^8–10^.

Bi_2_Te_3_ and its alloy are the best TE materials near room temperature, which are anisotropic with a layered structure. Their thermal and electrical conductivities along the *a*-axis (in the *c*-plane) are approximately two and four times higher, respectively, than those along the c-axis of Bi_2_Te_3_-based materials. But Seebeck coefficients are less dependent on the crystallography^11–14^. Therefore, an improved *ZT* value can be expected when utilizing the anisotropic thermal and electrical transport properties. Our previous results show that the hierarchical Sb_2_Te_3_ pillar array structure can selectively scatter phonon more than carrier, leading to a high *ZT* value^15^. In addition, we also find that unique nanowire array structuring can induce a change of the Fermi level of the Bi_2_(Te,Se)_3_ and favorably influence the phonon and carrier transport, thus dramatically enhancing a *ZT* result^16^. The previous studies have witnessed the feasibility of controlling novel microstructures to modify TE properties of Sb_2_Te_3_ and Bi_2_Te_3_-based alloys. However, it is noted that the hierarchical Sb_2_Te_3_ pillar array and the Bi_2_(Te,Se)_3_ nanowire array are grown perpendicular to the substrates and possess relatively high densities of interspaces, which degrade the in-plane transport properties to some extent. Stranz and Sun reported that a wafer-scale vertical nanopillar arrays or nanowire arrays can be realized by lithography and anisotropic etching for improving the performance of TE cross-plane devices as proposed recently^17,18^. But it is hardly to overcome a problem of carrier and phonon transport along the in-plane direction and measure cross-plane TE properties for vertical nanopillar arrays or nanowire arrays films. These adverse factors need to be further improved in films, enabling films to show better in-plane properties. Some vertically aligned nanowire arrays or nanopillar arrays have been synthesized by the electrochemical deposition with templates or the anisotropic etching and lithography method, however, this kind of hierarchical nanopillar arrays with tilt-structure have never been reported, let alone the tilted Bi_1.5_Sb_0.5_Te_3_ material. This motivates us to further explore the effect of tilt-structure on the hierarchical Bi_1.5_Sb_0.5_Te_3_ nanopillar arrays.

Hence, in this work, we aim to control the tilt angle of hierarchical Bi_1.5_Sb_0.5_Te_3_ nanopillar arrays based on the construction of one-dimensional nanowires. A simple thermal evaporation technique without using any templates was carried out, to our best knowledge, for the first time on the tilt-growth of hierarchical Bi_1.5_Sb_0.5_Te_3_ nanopillar arrays. The unusual multi-scale and multi-dimension structure Bi_1.5_Sb_0.5_Te_3_ nanopillar array with a tilt angle of 45° exhibits a stringkly high in-plane *ZT* = 1.61 at room temperature. It is believed that the interrelationship between the tilt-structure growth of films and the tilt angle of deposition substrates uncovered by this work may help to better understand unique tilt structuring of this kind of material. Furthermore, it provides a new avenue to control the structural configuration of materials with possible relevance to improvement of their properties.

## Results and Discussion

**Figure 1:**
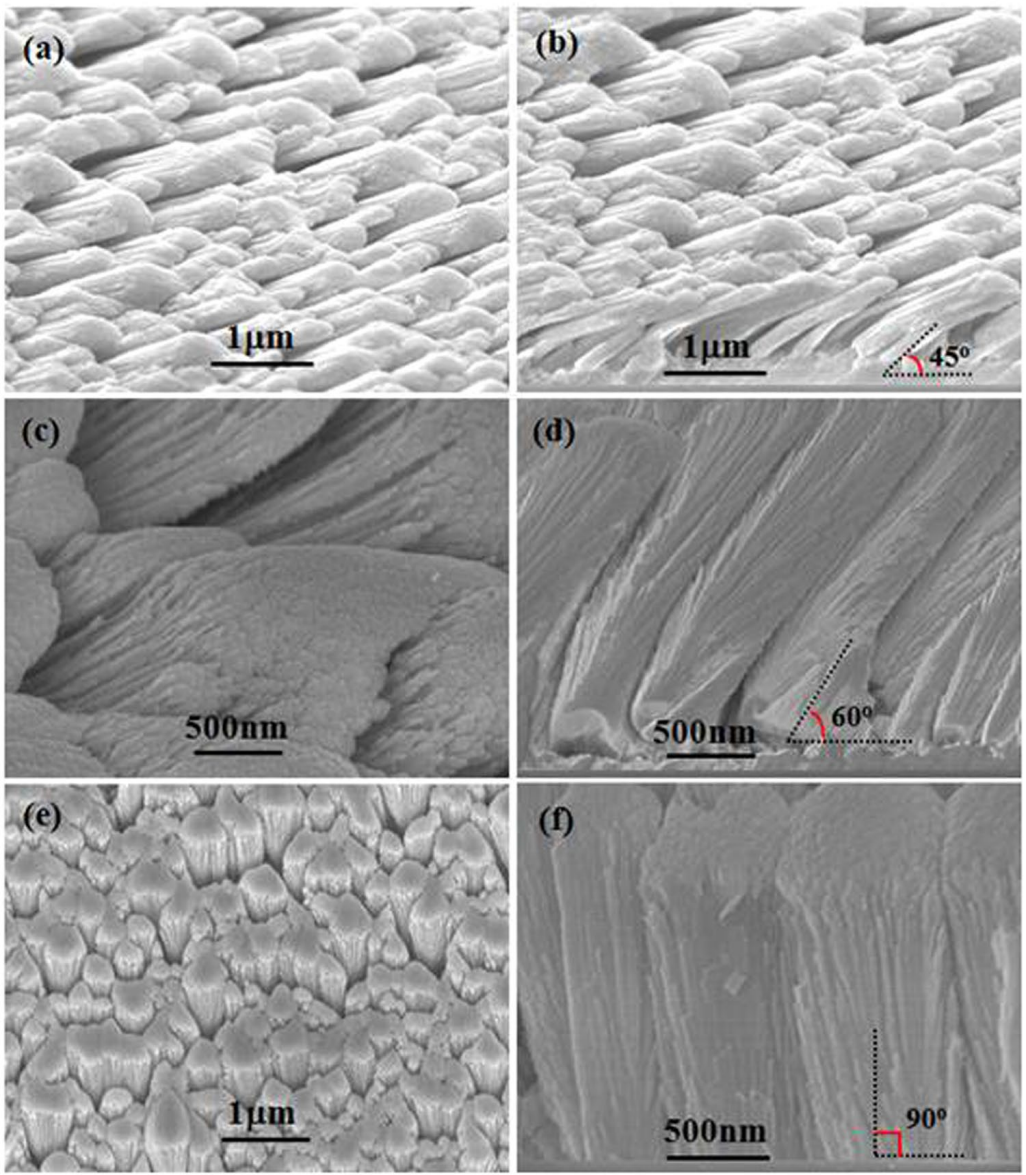
SEM images of the surface (**a**,**c**,**e**) and cross section (**b**,**d**,**f**) of hierarchical Bi_1.5_Sb_0.5_Te_3_ nanopillar arrays with tilt angles of (**a**,**b**) 45°, (**c**,**d**) 60° and (**e**,**f**) 90°.

The morphologies of hierarchical Bi_1.5_Sb_0.5_Te_3_ nanopillar arrays with tilt angles of 45°, 60°, and 90° were studied by SEM, respectively. The SEM images (Fig. 1a,b) reveal that the hierarchical Bi_1.5_Sb_0.5_Te_3_ nanopillar array with a tilt angle of 45° has been perfectly prepared by a simple thermal evaporation technique, which indicates a tilted growth when the tilt angle of the substrate plane to the horizontal plane is 45°. Seen from the top view (Fig. 1a), the hierarchical tilt-structure film is composed of nanopillar arrays based on the construction of one-dimensional nanowires. The diameters of tilt-growth nanowires are estimated to be <20 nm, implying a large number of unique interfaces in the nanopillar array. Many interspaces between nanopillar arrays are found, but the adjacent nanopillars have been tilted and interconnected closely to give a very good contact each other, guaranteeing carriers transport in the in-plane direction of the film. Seen from the cross-sectional image of the hierarchical film (Fig. 1b), a large number of Bi_1.5_Sb_0.5_Te_3_ nanowires are densely grown tilted to the substrate, along their preferred growth direction. Each nanopillar is formed by the assembly of lots of nanowires. It clearly shows that the tilt angle of the radial direction of nanopillars to the substrate plane is about 45°. A more condensed Bi_1.5_Sb_0.5_Te_3_ thin layer is formed near the substrate. By controlling growth parameter, nanopillar arrays microstructures have obviously changed as shown in Fig. 1. When the tilt angle of the substrate plane to the horizontal plane is 30°, the hierarchical Bi_1.5_Sb_0.5_Te_3_ nanopillar array with a tilt angle of 60° has been successfully fabricated (Fig. 1c,d). Seen from the surface SEM image (Fig. 1c), some nano-scaled open gaps between nanopillars can be found in the nanopillar array. From Fig. 1d, we note that numerous nanowires are tilted growth on the substrate and the tilt angle of the radial direction of nanopillars to the substrate plane is approximately 60°. Also, a thin layer is appeared near the substrate. These condensed thin layers could act as the seed crystal layers to induce the growth of nanopillar arrays with tilt angles of 45° and 60°, respectively. With the angle between the substrate plane and the horizontal plane reduces to about 0°, that is, the substrate approximatively parallels to the horizontal plane. The nanopillar array is densely grown perpendicular to the substrate, which exhibits that the angle of the radial direction of vertical nanopillars to the substrate plane is about 90° (Fig. 1e,f). Different from the above result, a thin layer is not appeared near the substrate. This phenomenon is the same to the reported result for the vertically aligned Sb_2_Te_3_ pillar array with hierarchical structure^15^.

**Figure 2:**
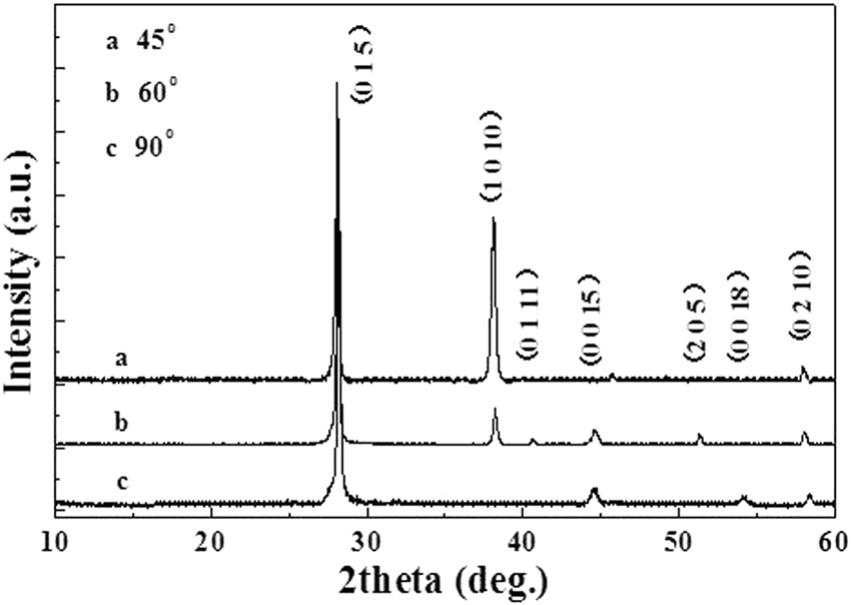
XRD patterns of hierarchical Bi_1.5_Sb_0.5_Te_3_ nanopillar arrays with tilt angles of 45°, 60° and 90°.

In order to gain insight into the crystal structure, nanopillar arrays were examined by XRD. Figure 2 presents XRD patterns of hierarchical Bi_1.5_Sb_0.5_Te_3_ nanopillar arrays with tilt angles of 45°, 60°, and 90°. As shown in Fig. 2, a single Bi_1.5_Sb_0.5_Te_3_ phase, consistent with the standard card (JCPDS 49–1713) of the Bi_1.5_Sb_0.5_Te_3_, was obtained in hierarchical nanopillar arrays samples. A preferential orientation (0 1 5) peak was mainly observed in all nanopillar arrays. The intensity of (1 0 10) texture of the hierarchical nanopillar array with a tilt angle of 45° is dramatically strong. When the tilt angle becomes large to 60°, the intensity of (1 0 10) peak of the hierarchical nanopillar array becomes weak. Finally, the peak is disappeared in the hierarchical nanopillar array with a tilt angle of 90°. This seems to indicate that the tilt angles of the nanopillars to the substrate are associated with the (1 0 10) peak of nanopillar arrays. The atom lateral mobility increase on the surface due to a decrease in the angle of the deposition direction to the substrate plane may be responsible for the structure change. The growing grains can be sufficiently mobile to migrate to the preferred sites for crystallization growth.

**Figure 3:**
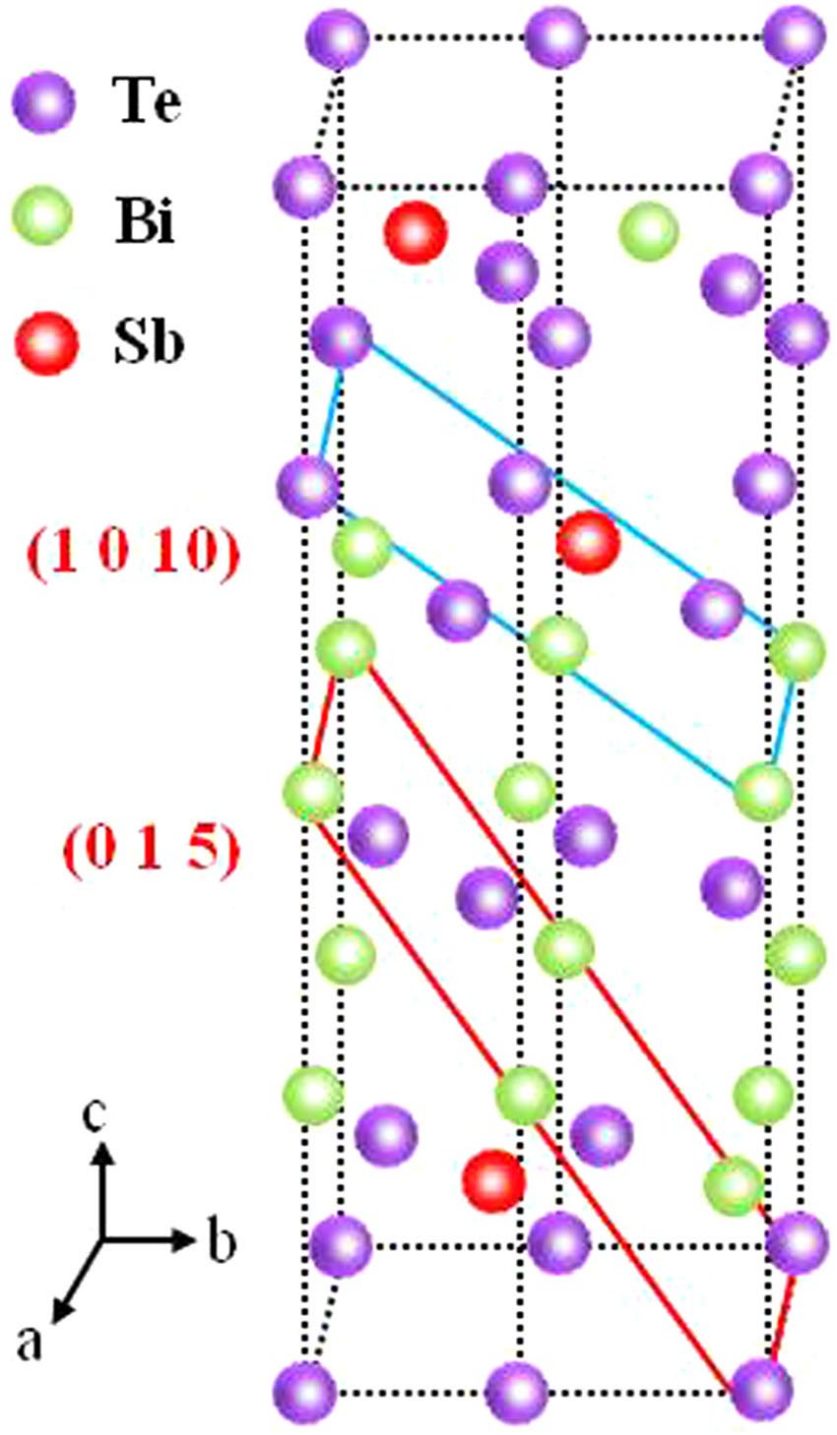
Theoretically simulated model of crystal structure of Bi_1.5_Sb_0.5_Te_3_.

Then the simulated model of Bi_1.5_Sb_0.5_Te_3_ molecular structure was built through Vestal to further explore the deposition mechanism of the Bi_1.5_Sb_0.5_Te_3_ films. Figure 3 illustrates the cell structure of Bi_1.5_Sb_0.5_Te_3_ molecular. The structure of Bi_1.5_Sb_0.5_Te_3_ is similar to that of Bi_2_Te_3_. In Bi_1.5_Sb_0.5_Te_3_ crystal, a quarter of Bi sites are occupied by Sb atoms. The (0 1 5) and (1 0 10) preferential orientations are depicted in the structure. As seen from Figs 1 and 2, these microstructural evolutions clearly indicate that decreasing the angle between the deposition direction and the substrate plane can not only help to form the (0 1 5) and (1 0 10) preferential orientations but also control the growth of Bi_1.5_Sb_0.5_Te_3_ nanopillar arrays. Thin films of PVD deposited materials normally grow with the plane of highest atomic density or the plane with minimum surface free energy^19–21^. The (0 1 5) and (1 0 10) planes perhaps have relatively low surface free energy. As can be seen from Fig. 3, since the surface atom density of the (0 1 5) and (1 0 10) planes is the relatively large, the surface free energy of the (0 1 5) and (1 0 10)-oriented film should be the small according to Gibbs-Wulff theorem. Besides, an explanation of the selection of the (0 1 5) and (1 0 10) preferred orientations by the incident ion beams, based on the ion sputtering effect, is suggested. For those (0 1 5) and (1 0 10) grains, their [0 1 5] and [1 0 10] directions are not facing the incident beams. Therefore, these grains experience less ion damage when the tilt angle of the deposition direction to the substrate plane is 45°. However, the angle of the deposition direction to the substrate plane is 60° or 90°, (1 0 10) grains possibly experience much ion damage. The beneficial roles of the preferential orientations in improving transport properties are greatly important for the Bi_1.5_Sb_0.5_Te_3_ films.

**Figure 4:**
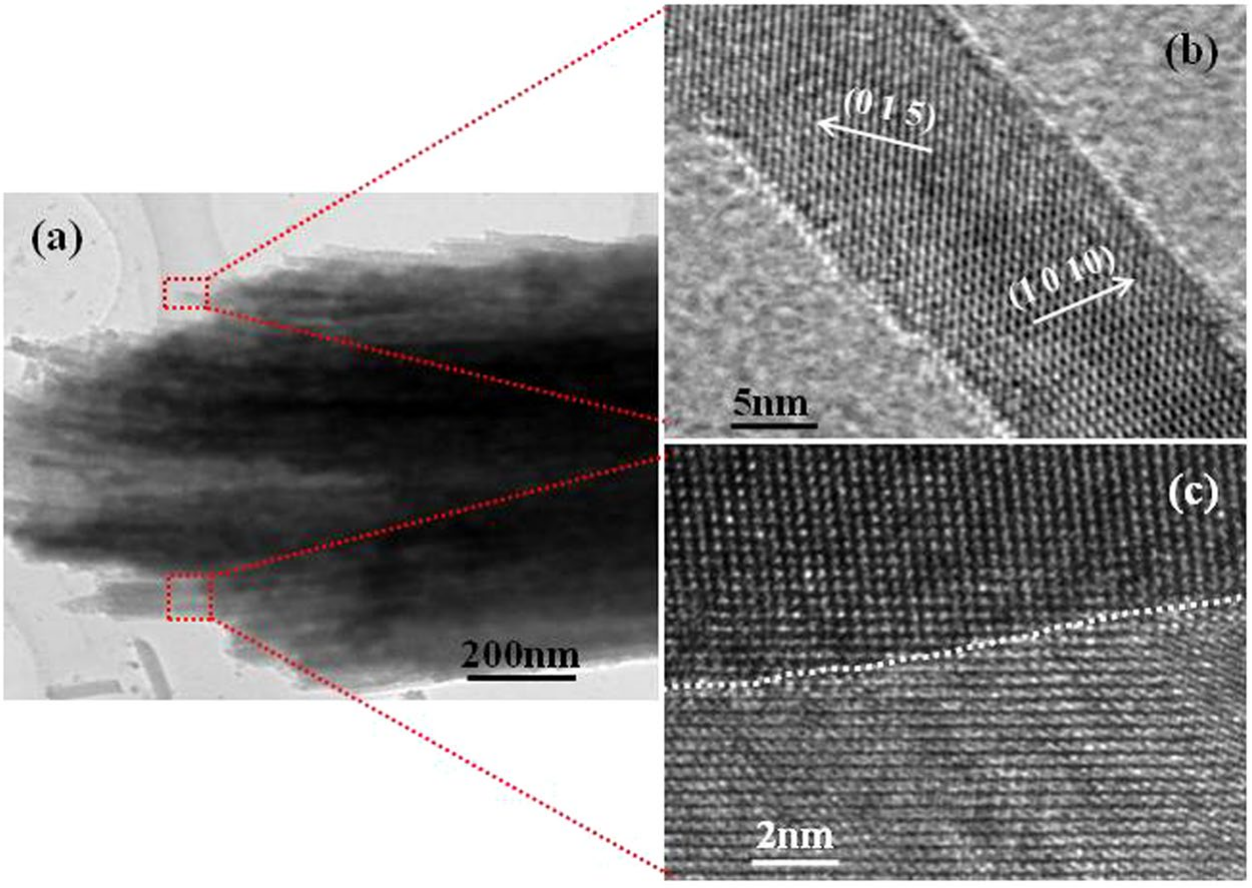
TEM and HRTEM images of the hierarchical Bi_1.5_Sb_0.5_Te_3_ nanopillar array with a tilt angle of 45°. (**a**) The enlarged image of the nanopillar array; (**b**,**c**) images of selected area respectively marked by a square in (**a**). (The interface between nanowires marked by the dot line, as shown in (**c**)).

The details of the specially hierarchical nanopillar array microstructure are observed in TEM and HRTEM images, as depicted in Fig. 4. A hierarchical microstructure of Bi_1.5_Sb_0.5_Te_3_ nanopillars with a tilt angle of 45° varies continuously from the nanowire surface to the unique interface shown in Fig. 4a, which can significantly influence the carrier transport. It clearly shows that nanowires tightly connect side-by-side to assemble into nanopillars, which would be necessary to improve the transport property by preserving the high quality channel region from the ordered nanowire region through the alignment of interfaces. The lattices of (0 1 5) and (1 0 10) crystal planes are exhibitted in Fig. 4b which originates from the magnified image of the selected area in Fig. 4a. It shows that the nanowires grow along the preferred [0 1 5] and [1 0 10] directions. The diameter of nanowires is confirmed to be <20 nm, and nanowires show rough surfaces which can greatly increase phonon scattering. Furthermore, the interface between nanowires remains substantially coherent, as shown in Fig. 4c. Thus, while phonons are strongly scattered, the carrier transport is only little impeded in the nanopillar array. This microstructure would play an extremely positive role on its TE properties.

**Figure 5:**
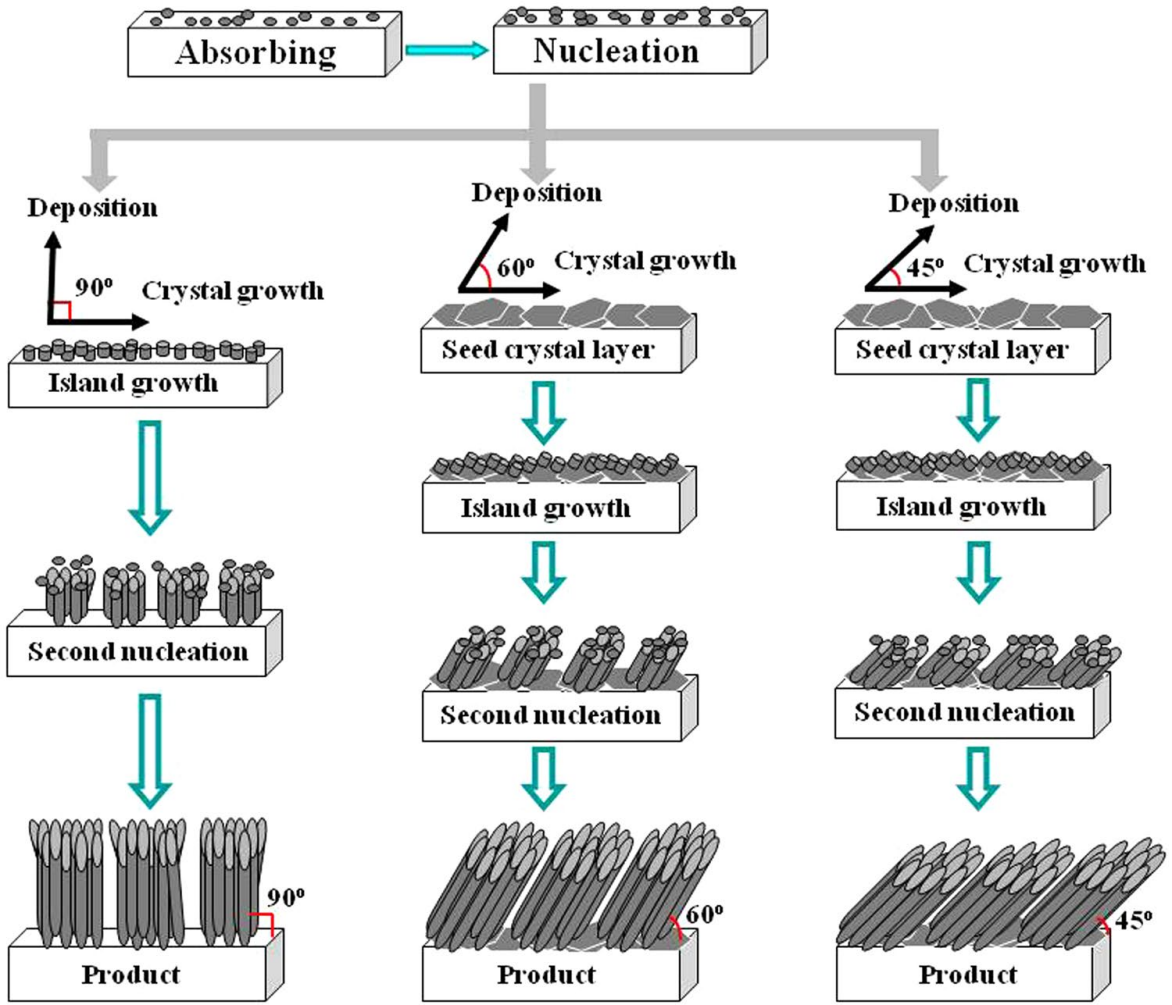
The growth mechanism of hierarchical Bi_1.5_Sb_0.5_Te_3_ nanopillar arrays with tilt angles of 45°, 60° and 90° in different deposition stages.

The detailed growth mechanism of hierarchical Bi_1.5_Sb_0.5_Te_3_ nanopillar arrays with tilt angles of 45°, 60° and 90° will be proposed and further investigated, respectively. The growth process of nanopillar arrays are illustrated in Fig. 5, which are explained by theoretical models^22^. Three primary modes of thin-film growth include: (1) Volmer-Weber (3D island formation), (2) Frank-van der Merwe (2D layer-by-layer), (3) Stranski-Krastanov (layer-plus-island), Stranski-Krastanov growth is an intermediary process characterized by both 2D layer and 3D island growth. Theoretically, neglecting the strain energy of films, the growth modes of thin films are determined by the free energy of the substrate surface (*σ*_s_), the surface free energy of the heteroepitaxial layer (*σ*_h_), and the interface free energy (*σ*_i_). When the angle of the substrate plane to the horizontal plane is about 0°, that is, the substrate parallels to the horizontal plane, and the deposition direction is approximatively vertical to the substrate. In the initial nucleation stage, the inequality *σ*_s_ <*σ*_i_ + *σ*_h_ sets the condition for the epitaxial film, no wetting the substrate, which represents the Volmer-Weber growth. Thermal evaporation produces relatively high energy depositing particles due to which enhanced number of nucleation sites is produced, leading to fine grains and small island growth. Subsequently, a second nucleation and growth process happened on the already formed 3D islands, and the preferential direction of 3D islands is (0 1 5) (SEM images of (0 1 5)-preferential 3D islands are provided in Supporting Information, Figure S2). The stabilized fast flux of incoming particles of thermal evaporation restricts the surface diffusion of adatoms resulting in the formation of small nanowire arrays morphology. Simultaneously, the surface dangling bonds on nanowires and the surface energy and stress energy etc. are also responsible for the formation of the vertically aligned Bi_1.5_Sb_0.5_Te_3_nanopillar array with hierarchical structure, which is similar to our previous result for the vertical Sb_2_Te_3_ pillar array with hierarchical structure^15^. With increasing the angle between the substrate plane and the horizontal plane to 30°, the angle of the deposition direction to the substrate plane is 60°. In this case, the deposited atoms or ions can obtain some energy for slightly lateral movement on the substrate surface, being equivalent to increase *σ*_s_. The inequality *σ*_s_ >*σ*_i_ + *σ*_h_ sets the condition for the epitaxial film to wet the substrate. The 2D layer growth occurs and the seed crystal layer is formed. Next, atoms or ions are deposited on the surface of the seed crystal layer. As the layer thickness increases, the accumulated thermal stress and strain energy contribute to *σ*_i_. Then, the tilted 3D island growth occurs (*σ*_s_ <*σ*_i_ + *σ*_h_). The growth process happened on the already formed seed crystal layer, and the oriented direction of the seed crystal layer and tilt-structure 3D islands is [0 1 5] and [1 0 10]. Subsequently, a second nucleation and growth process happened on the already formed tilt-structure 3D islands. Under this condition, the tilt-structure crystal growth in the radial direction is faster than that of the planar direction. Thus, under the induced effect of (0 1 5) and (1 0 10) lattice planes, the key for fabricating the hierarchical Bi_1.5_Sb_0.5_Te_3_ nanopillar array with a tilt angle of 60° is to keep the relative high deposition rate to grow in the radial direction. Therefore, the angle of 60° between the deposition direction and the substrate plane is necessary experiment condition for fabricating the hierarchical Bi_1.5_Sb_0.5_Te_3_ nanopillar array with a tilt angle of 60°. When the angle between the substrate plane and the horizontal plane increases to 45°, that is, the angle of the deposition direction to the substrate plane is 45° (see Fig. 5), the growth process for the hierarchical Bi_1.5_Sb_0.5_Te_3_ nanopillar array with a tilt angle of 45° is very similar to that of the nanopillar array with a tilt angle of 60° in a Stranski-Krastanov mode.

**Figure 6:**
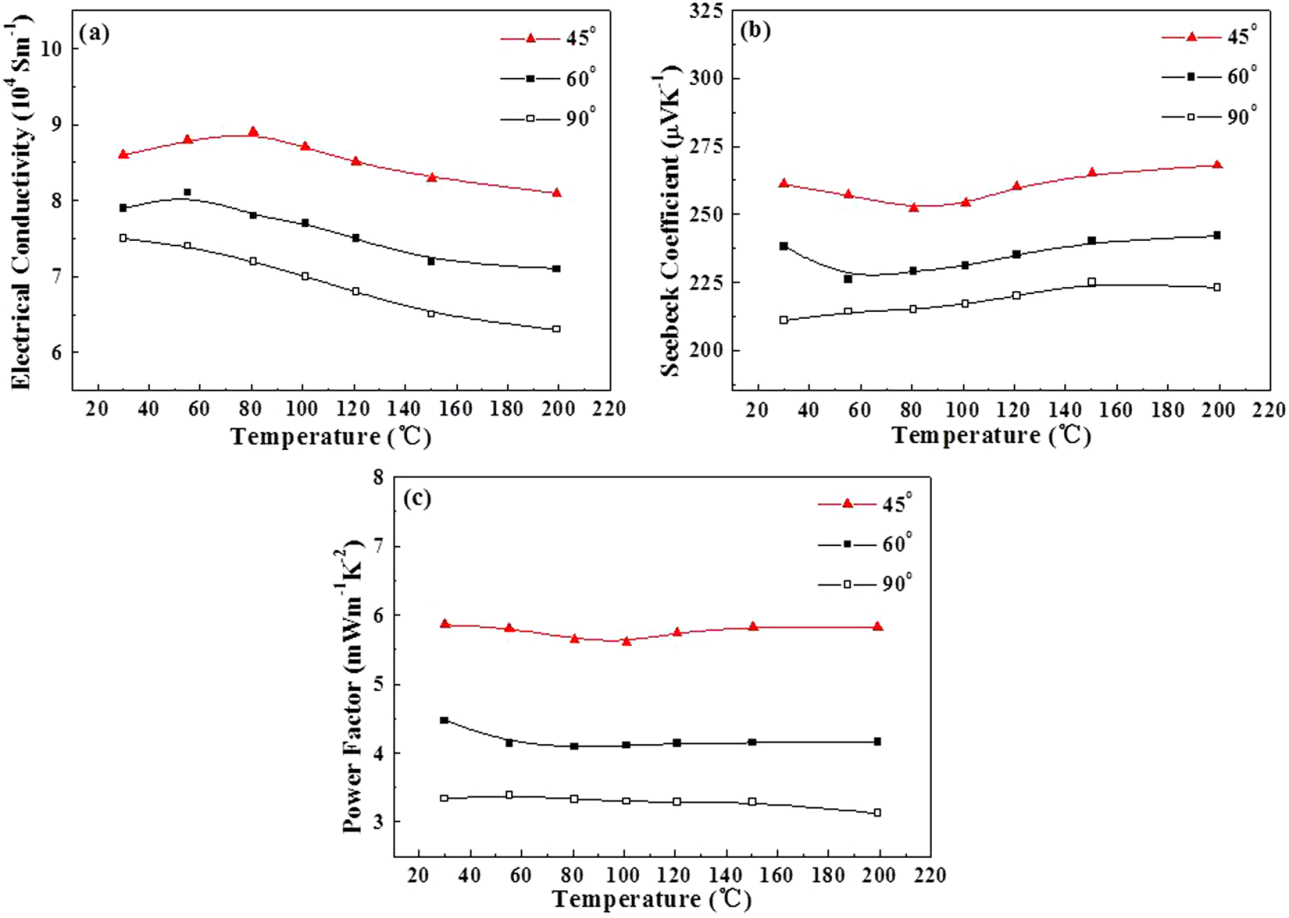
(**a**) Electrical conductivities, (**b**) Seebeck coefficients, and (**c**) power factors of hierarchical Bi_1.5_Sb_0.5_Te_3_ nanopillar arrays as a function of temperature.

It is of considerable interest to be able to tune electronic transport properties by modifying the morphology and crystal structure of films. The in-plane electrical conductivities of hierarchical Bi_1.5_Sb_0.5_Te_3_ nanopillar arrays with tilt angles of 45°, 60° and 90° were investigated, respectively. As shown in Fig. 6a, nanopillar arrays with tilt angles of 45°, 60° and 90° have maximum electrical conductivities of 8.9 × 10^4^ S m^−1^, 8.1 × 10^4^ S m^−1^ and 7.5 × 10^4^ S m^−1^ in the temperature range of 30–200 °C, respectively, which is higher than those obtained from materials^12,23–25^. We can obviously see that the in-plane electrical conductivities of nanopillar arrays increase as the tilt angles of nanopillar arrays decrease. The enhanced electrical conductivities in nanopillar arrays are thought to be mainly related to unique tilt-structure of nanopillar arrays. Besides, the Bi_1.5_Sb_0.5_Te_3_ material is a well-known narrow band gap semiconductor, there are surface states at an energetic position above the conduction band edge, which leads to a charge transfer from the surface state into the bulk^26^. Thus, an electron layer accumulates at the surface region of Bi_1.5_Sb_0.5_Te_3_ nanowires with a high surface-to-volume ratio, leading to significantly increased electrical conductivities. Moreover, it may be speculated that the strong oriented (0 1 5) and (1 0 10) lattices and tilt-structure of nanopillar arrays provides the fastest channels for carriers transport. This gives a reasonable explanation why the carrier mobility and electrical conductivity were enhanced in the nanopillar array with a tilt angle of 45°.

In Fig. 6b, the temperature dependent Seebeck coefficients of hierarchical Bi_1.5_Sb_0.5_Te_3_ nanopillar arrays with tilt angles of 45°, 60° and 90° are presented, respectively, which are positive values for all samples, indicating a *p*-type semiconductor. It shows that the highest Seebeck coefficient reaches to 268 μV K^−1^ for the hierarchical nanopillar array with a tilt angle of 45° at the temperature of 200 °C. An outstanding increase in the Seebeck coefficient is observed with a decrease in tilt angles for the hierarchical nanopillar arrays. The Seebeck coefficient value in the present work is much higher in comparison to the reported results of bismuth antimony telluride materials^12,23–25,27–31^. The reason for this greatly difference is the change in the microstructure compared to the reported structure. This tilt-structure can possibly promote the carrier mobility in the in-plane direction, leading to large Seebeck coefficients.

The power factor *S*^2^*σ* versus temperature for hierarchical Bi_1.5_Sb_0.5_Te_3_ nanopillar arrays is plotted in Fig. 6c. The power factor values of the hierarchical Bi_1.5_Sb_0.5_Te_3_ nanopillar array with a tilt angle of 45° slightly decrease firstly and then increase with increasing temperature. It exhibits that a strikingly high average power factor of 5.76 mW/m·K^2^ was obtained for the unique tilt-structure film between 30–200 °C, along with the maximum power factor value of 5.86 mW/m·K^2^ at 30 °C, which also implies that the *S*^2^*σ* nearly remains constant with temperature. In contrast to Sb_2_Te_3_-based and Bi_2_Te_3_-based materials^12,23–25,27–31^, whose *S*^2^*σ* values increase or decrease as the temperature increases, the temperature stability of *S*^2^*σ* of the hierarchical Bi_1.5_Sb_0.5_Te_3_ nanopillar array with a tilt angle of 45° are useful in practical use. It is clearly demonstrated that the hierarchical Bi_1.5_Sb_0.5_Te_3_ nanopillar array with a tilt angle of 45° shows a largely enhanced power factor compared with the hierarchical nanopillar array with a tilt angle of 60° or 90° and previous results^29–32^. There is no doubt that the tilt architecture significantly aids in achieving high TE properties, especially for the hierarchical nanopillar array with a tilt angle of 45°. However, the novel structure material was further considered to have better TE properties due to the greatly reduce thermal conductivities originating from lots of interfaces, numerous interspaces, multi-scaled grains, etc.

The uniquely hierarchical film is composed of nanopillar arrays based on the construction of 1D nanowires, which is expected to be low in-plane *κ* values. The *κ* of the hierarchical Bi_1.5_Sb_0.5_Te_3_ nanopillar array with a tilt angle of 45° is 1.09 W m^−1^ K^−1^ (see Table 1), which is slightly larger than values of the nanopillar arrays with tilt angles of 60° and 90° (1.04 and 0.96 W m^−1^ K^−1^) due to the influence of the tilt-structure of nanopillar arrays. However, the presented thermal conductivities are lower than the reported results for Bi-Sb-Te materials^25,32,33^. The excellent phonon transport properties are attributed to the comprehensive effects induced by the multi-scaled grains, thin nanowires, nano-scaled open gaps, coherent grain boundaries, rough surfaces, surface dangling bonds, antisite defects as well as other defects. These factors together are responsible for scattering phonon with a variety of wavelengths, leading to low thermal conductivity in unique nanopillar arrays, as SEM and TEM microstructure above.

**Table 1:** Transport properties and compositions of hierarchical Bi_1.5_Sb_0.5_Te_3_ nanopillar arrays measured at room temperature.

Tilt angle	Bi/Sb/Te atomic ratio	Carrier concentration (10^19^/cm^3^)	Carrier mobility (cm^2^/V·s)	Electrical conductivity (10^4^ S/m)	Seebeck coefficient (μV/K)	Thermal conductivity (W/m·K)	*ZT* ~300 K
45^o^	29.8/10.0/60.2	4.2	130	8.6	261	1.09	1.61
60^o^	29.7/10.2/60.1	5.3	94	7.9	238	1.04	1.29
90^o^	29.7/10.1/60.2	6.7	69	7.5	211	0.96	1.04

According to the measured thermal conductivity, electrical conductivity, and Seebeck coefficient, the *ZT* value was calculated at room temperature, as shown in Table 1. The in-plane *ZT* of the hierarchical Bi_1.5_Sb_0.5_Te_3_ nanopillar array with a tilt angle of 45° was 1.61, which is superior to that of the nanopillar arrays with tilt angles of 60° and 90° and the reported results of Bi-Sb-Te film and bulk materials^12,15,16,23–25,28–34^, and comparable with the value (1.86) of the bulk Bi_1.5_Sb_0.5_Te_3_ material as reported recently^35^. Tilt-structuring opens great opportunities for an effective modification of the interrelated TE transport properties at hierarchical nanopillar arrays with tilt growth, where the size effect^36^, energy filtering effect^37^, and preferential route effect can have a profound influence on the TE transport properties. The different behavior of the hierarchical nanopillar array with tilt-structure in terms of thermal and electrical conduction strongly suggests that the hierarchical Bi_1.5_Sb_0.5_Te_3_ nanopillar arrays promise to be a most efficient structure for TE devices. The results of this study provide insights for the structural design and synthesis of TE materials, which will be very important for the development of functional materials in the future.

In order to further verify novel tilt-structure nanopillar arrays playing a greatly important role to optimize TE transport properties, the mobility and concentration of carriers were examined at room temperature. The composition analysis by EDX was at first considered due to the composition of films affecting the carrier concentration. The result of EDX confirms that the atomic ratios are quite similar to those of the Bi_1.5_Sb_0.5_Te_3_ powder in five different regions of each nanopillar array sample, as shown in Table 1. EDX compositional analyses indicate slightly Te rich in all Bi_1.5_Sb_0.5_Te_3_ films. Note that the carrier concentration of bismuth antimony telluride depends on acceptor-like Sb_Te_ (Bi_Te_) defects and donor-like V_Te_ vacancies. The Te-excess samples can suppress the generation of V_Te_ vacancies under a Te-rich condition. We believe that slightly Te-excess samples can provide an improvement in hole carrier concentration. It can be seen that the carrier mobility and carrier concentration exhibit different among these nanopillar arrays. The concentrations of carriers are 4.2 × 10^19^ cm^−3^, 5.3 × 10^19^ cm^−3^ and 6.7 × 10^19^ cm^−3^ for hierarchical Bi_1.5_Sb_0.5_Te_3_ nanopillar arrays with tilt angles of 45°, 60° and 90°, respectively. These films possess the value of carrier concentration 10^19^ cm^−3^. It is found that the optimal carrier concentration for the best power factor is approximately 10^19^ cm^−3^ in Bi-Sb-Te materials^38^. Besides, it clearly exhibits that the concentrations of carriers decrease owing to the formation of a relatively perfect crystalline structure at a proper deposition condition. Here, the hierarchical Bi_1.5_Sb_0.5_Te_3_ nanopillar arrays with an angle of 90° obtains the relatively high value of the carrier concentration possibly due to the formation of lots of defects, which negatively effects on Seebeck coefficients. On the other hand, the ordered nanopillar array with a tilt angle of 45° has a high mobility of 130 cm^2^/V·s, which is prominently higher than that of the nanopillar arrays with tilt angles of 60° and 90° (94 cm^2^/V·s and 69 cm^2^/V·s), respectively, as shown in Table 1. It shows that the unique 45° tilt angle structure is more beneficial for carrier mobility in comparison with 60° or 90° tilt angle structure, then enhancing power factors. It is a fact that the length and the surface area for the nanowires increase when tilt angles of films with the same thickness become small. The long nanowires can provide the large contact area for the adjacent nanowires, leading to the increase in the chance of carriers transport. Besides, the strong oriented (0 1 5) and (1 0 10) crystal planes are similar to two highways for carriers transport in the Bi_1.5_Sb_0.5_Te_3_ nanopillar arrays with a tilt angle of 45°. Therefore, it is easy to understand that the (0 1 5) and (1 0 10)-oriented structure can remarkably increase the carrier mobility and enhance power factors in nanopillar arrays. Taking advantage of optimization to structure, the enhanced TE performance has been achieved by the new avenue to adjust the carrier and phonon transport. It is believed that this direction of research will inspire a flurry of interest in exploring effective approaches to fabricate such hierarchical tilt-structure TE materials using scalable, simple, and controllable synthesis processes.

## Conclusions

Uniquely hierarchical Bi_1.5_Sb_0.5_Te_3_ nanopillar arrays with tilt angles of 45°, 60° and 90° based on the construction of one-dimensional nanowires have been fabricated by a simple thermal evaporation technique. The unusual multi-scale and multi-dimension structure Bi_1.5_Sb_0.5_Te_3_ nanopillar array with a tilt angle of 45° exhibits the highest Seebeck coefficientof 268 μV K^−1^ at 200 °C, the maximum power factor value of 5.86 mW/m·K^2^ at 30 °C, the average power factor of 5.76 mW/m·K^2^ between 30–200 °C, along with the strikingly high thermoelectric performance *ZT* = 1.61 at room temperature. The strong preferred (0 1 5) and (1 0 10) lattices and tilt-structure of nanopillar arrays provides the fastest channels for carriers transport, enhancing TE properties in the nanopillar array with a tilt angle of 45°. With optimizing tilt angles, crystal orientation, nanowires diameter, nano-scaled open gaps, and coherent surfaces, the TE performance is likely to be better. It provides a new control over the structural configuration of materials with relevance to improvement of their properties.

## Methods

In this work, the hierarchical Bi_1.5_Sb_0.5_Te_3_ nanopillar arrays with tilt-structure can be prepared by a simple vacuum thermal evaporation technique. A sketch of the vacuum thermal evaporation setup is provided in Supporting Information, as shown in Figure S1. The angle between the substrate plane and the horizontal plane can be controlled by adjusting the substrate holder. In order to successfully grow the hierarchical Bi_1.5_Sb_0.5_Te_3_ nanopillar arrays with tilt angles of 45°, 60°, and 90° on SiO_2_ glass substrates by the thermal evaporation technique, the corresponding angles between the substrate holder plane and the horizontal plane are supposed to be about 45°, 30°, and 0°, respectively. Due to Te deficiency easily occurred in Bi_2_Te_3_-based films, Te and Bi_1.5_Sb_0.5_Te_3_ powders (99.99% purity, the mass rate of Te:Bi_1.5_Sb_0.5_Te_3_ is about 1:10) were put on the evaporating dish, and the evaporated current was 165 A for all hierarchical nanopillar arrays. Before deposition, the SiO_2_ substrate was at first cleaned by diluted nitric acid, and then acetone, and dried under the nitrogen gas flow. Next, the SiO_2_ substrate was loaded onto the sample holder. In ordered to remove oxygen, N_2_ gas was introduced into the chamber and vacuumized three times. The deposition temperature was set at 300 °C, the working pressure was maintained at 2 × 10^−6^ Torr in the film deposition process. The thickness of films could be controlled by changing the deposition time in our experiments.

The crystal structure characterizations for the nanopillar arrays were measured using x-ray diffraction (XRD) on a Rigaku D/MAX 2200 PC automatic x-ray diffraction with Cu K_α_ radiation (*λ* = 0.154056 nm). A field emission scanning electron microscope (FE-SEM, Sirion 200) equipped with an energy dispersive x-ray spectroscope (EDX) were used to investigate the morphology and composition of all samples. The crystal structure of Bi_1.5_Sb_0.5_Te_3_ nanopillar array was performed with the aid of a high-resolution transmission electron microscope (HRTEM) (Tecnai G2 F20S-Twin). The film thickness was measured by surface profilometry (Ambios XP-2, USA). The Seebeck coefficients and electrical conductivities of all films were simultaneously examined by ZEM-3 (Ulvac Riko, Inc.). A Laser PIT (Ulvac Riko, Inc.) was used to collect the in-plane thermal conductivity data at room temperature. The principle of the measurement method for the thermal conductivity is described in ref.^39^. The carrier mobility and carrier concentration were determined by a four-probe method using Hall effects measurement system (ECOPIA HMS-3000) at room temperature. All tests for transport properties were repeated at least 5 times. The errors are 5% for thermal conductivities, 4% for electrical conductivities, 5% for Seebeck coefficients, and 10% for *ZT* values.

## Electronic supplementary material

Tilt-structure and high-performance of hierarchical Bi1.5Sb0.5Te3 nanopillar arrays
