# Supplementary material for: Tilt-structure and high-performance of hierarchical Bi1.5Sb0.5Te3 nanopillar arrays
Source: Sci Rep. 2018 Apr 23;8:6384. doi: 10.1038/s41598-018-24872-4 (PMC5913231; doi:10.1038/s41598-018-24872-4)
Supplement: Supplementary file 1 — Tilt-structure and high-performance of hierarchical Bi1.5Sb0.5Te3 nanopillar arrays [file 41598_2018_24872_MOESM1_ESM.doc]

**Supporting Information**

**Tilt-structure and high-performance of hierarchical Bi_1.5_Sb_0.5_Te_3_ nanopillar arrays**

Ming Tan, Yanming Hao, Yuan Deng, Dali Yan, Zehua Wu


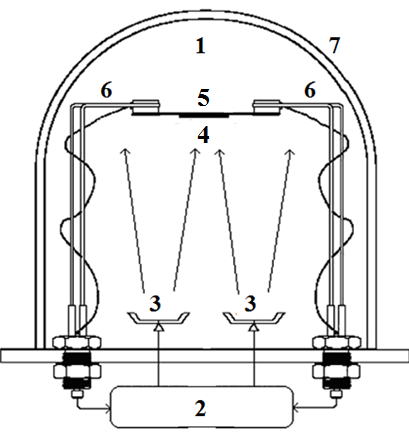


**Fig S1** A sketch of vacuum thermal evaporation setup

(1 Vacuum chamber, 2 PID controller, 3 Tungsten boat, 4 Substrate, 5 Sample holder, 6 Film thickness detector, 7 Bell glass)


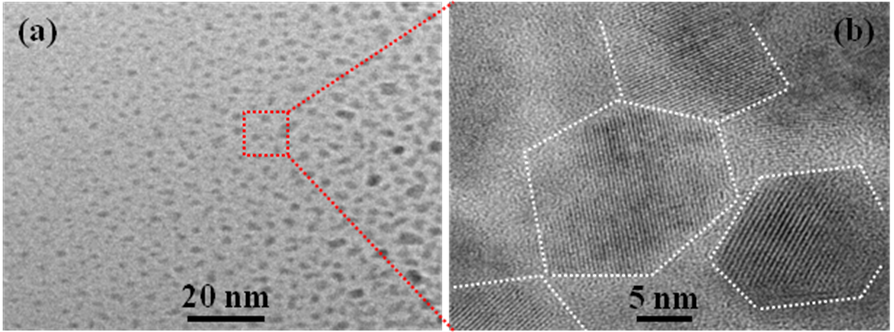


**Fig S2** The (0 1 5)-preferential 3D islands in film growth process
